# The Role of Endoplasmic Reticulum Stress Response in Pollen Development and Heat Stress Tolerance

**DOI:** 10.3389/fpls.2021.661062

**Published:** 2021-04-14

**Authors:** Mohan B. Singh, Neeta Lohani, Prem L. Bhalla

**Affiliations:** Plant Molecular Biology and Biotechnology Laboratory, Faculty of Veterinary and Agricultural Sciences, The University of Melbourne, Parkville, VIC, Australia

**Keywords:** endoplasmic reticulum stress, unfolded protein response, plant reproduction, pollen development, male gametophyte, heat stress, pollen, sperm cell

## Abstract

Endoplasmic reticulum (ER) stress is defined by a protracted disruption in protein folding and accumulation of unfolded or misfolded proteins in the ER. This accumulation of unfolded proteins can result from excessive demands on the protein folding machinery triggered by environmental and cellular stresses such as nutrient deficiencies, oxidative stress, pathogens, and heat. The cell responds to ER stress by activating a protective pathway termed unfolded protein response (UPR), which comprises cellular mechanisms targeted to maintain cellular homeostasis by increasing the ER’s protein folding capacity. The UPR is especially significant for plants as being sessile requires them to adapt to multiple environmental stresses. While multiple stresses trigger the UPR at the vegetative stage, it appears to be active constitutively in the anthers of unstressed plants. Transcriptome analysis reveals significant upregulation of ER stress-related transcripts in diploid meiocytes and haploid microspores. Interestingly, several ER stress-related genes are specifically upregulated in the sperm cells. The analysis of gene knockout mutants in Arabidopsis has revealed that defects in ER stress response lead to the failure of normal pollen development and enhanced susceptibility of male gametophyte to heat stress conditions. In this mini-review, we provide an overview of the role of ER stress and UPR in pollen development and its protective roles in maintaining male fertility under heat stress conditions.

## Introduction

The endoplasmic reticulum (ER) is a large, structurally complex organelle whose membrane can constitute half of a eukaryotic cell’s total membranes. ER is a main production site for lipids and many proteins. Each cell carries two types of the ER: smooth ER (SER) and rough ER (RER). The SER is a site of lipid and sterol biosynthesis. In contrast, RER with its outer cytosol-facing surface studded with ribosome plays a crucial role in biosynthesis and productive post-translational processing and folding of secretory and transmembrane proteins. Nearly one-third of protein production and folding occurs in this organelle (Schubert et al., 2000). This highly active process requires finely tuned regulation of ER homeostasis. The protein homeostasis in the ER is maintained by chaperone functioning, folding, quality control (QC), and degradation systems. Following assembly on membrane-bound ribosomes, the unfolded polypeptides enter into the ER lumen for a chaperone-assisted folding to their correct three-dimensional conformation (Hetz et al., 2020) to enable them to perform their assigned biological functions. Other post-translational modifications in the ER include N-linked glycosylation and disulfide bond formation. Proteins that get folded successfully leave the ER and move towards their final destination through the secretory pathway.

**FIGURE 1:**
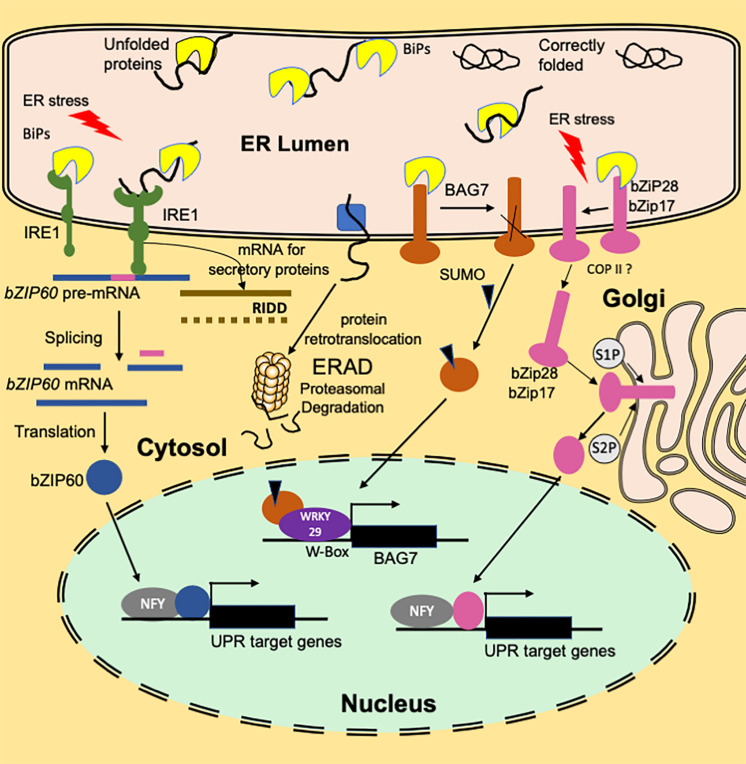
An overview of the functioning of plant ER stress signaling in response to heat stress. A branch of the UPR signaling pathway involves ER transmembrane sensor, IRE1, that propagates the UPR signal from ER to the cytosol. Association with luminal BIP keeps IRE1 in an inactive monomer form. Under ER stress conditions, BIP dissociates from the sensor end of IRE1 to facilitate the folding of the accumulated unfolded proteins. The binding of unfolded proteins to the luminal domain of IRE1 triggers dimerization (or oligomerization) and activation of RNase activity that cleaves bZIP60(u) mRNA resulting in a spliced variant bZIP60(s). Translation of the spliced variant leads to the synthesis of active bZIP60 TF protein whose transport to nucleus activates the stress-responsive genes. Another function of IRE1 is IRE1-Dependent RNA Decay (RIDD) that involves degradation of ribosome-associated RNAs encoding secretory proteins. Dissociation of BIP from ER-anchored transcription factors bZIP28/17 results in their mobilization to Golgi. In the Golgi, these TFs are processed to bZIP17(p) and bZIP28 (p) by S1P and S2P proteases to release cytosolic facing domains that are further transported to the nucleus. In the nucleus, bZIP28/17 bind to ER stress response elements to upregulate the transcription of UPR genes. Another branch of UPR involves an ER-resident transcription factor, BAG7. BAG7 is involved in UPR in response to heat and cold stress conditions by acting as a co-chaperone to prevent the accumulation of unfolded proteins. Under heat stress conditions, BAG7 is sumoylated, released from ER by protease and then translocated to the nucleus where it interacts with WRKY29 to regulate *BAG7* and other chaperone expression.

The protein folding is an intrinsically error-prone process with nearly 30% of the newly synthesized protein folded inappropriately (Balchin et al., 2016). When the folding fails, misfolded polypeptides are retained in the ER by QC mechanisms (Figure 1). The terminally misfolded and aggregated proteins are retrotranslocated into the cytosol to be degraded by endoplasmic-reticulum-associated degradation (ERAD) machinery (Li et al., 2017a). ERAD is an essential component of the ER QC system that clears toxic misfolded proteins via an ER-specific ubiquitin/proteasome system involving ubiquitin-activating enzyme (E1), ubiquitin-conjugating enzyme (E2), ubiquitin ligase (E3), and 26S proteasome system. Selective autophagy of ER termed ER phagy, which includes vacuolar degradation of cytoplasmic components, is another component of the ER QC process. Activation of autophagy leads to the *de novo* formation of double-membrane vesicles termed as autophagosomes at the ER that envelop damaged or superfluous cell components and traffic them to vacuoles for degradation to simple molecules for recycling them back into the cytosol (Wirawan et al., 2012; Marshall and Vierstra, 2018; Bao and Bassham, 2020).

Endoplasmic reticulum protein folding, export and degradation machinery can manage the protein folding demands under the homeostatic state. However, under certain physiological conditions or environmental stresses, the ER gets overwhelmed with misfolded proteins leading to ER stress (Figure 1). To cope with the ER stress, the ER triggers an adaptive program, the unfolded protein response (UPR) (Buchberger et al., 2010; Liu and Howell, 2010; Angelos et al., 2017). In contrast, a cytosolic protein response (CPR) involving specific heat shock factors functions to maintain protein homeostasis in the cytosol (Sugio et al., 2009). The CPR involves heat shock caused activation HSFs leading to enhanced expression of genes encoding heat shock proteins (HSPs). HSPs function as molecular chaperones to counteract protein aggregation and target misfolded proteins for degradation (Buchberger et al., 2010; Hartl et al., 2011; Li et al., 2017a). The compartmentalization of CPR and the ER UPR, with their own distinct chaperones, permits independent responses to disorders in protein folding processes.

The UPR response signature is enhanced expression of genes encoding ER chaperones and the components of the ERAD system (Martínez and Chrispeels, 2003). The UPR activation to prevent the accumulation of unfolded or misfolded proteins in the ER includes ER-localized sensor protein inositol requiring enzyme 1 (IRE1) (Deng et al., 2011). IRE1 functions as an RNA splicing factor whose principal substrate in plants is mRNA encoding the transcription factor bZIP60. Upon its activation by ER stress, IRE1 splices bZIP60 mRNA and produces a form that encodes a bZIP60 protein lacking transmembrane domain (Li and Howell, 2021). Another arm of the UPR pathway involves stress-induced membrane-associated transcription factors, bZIP17 and bZIP28. Molecular chaperones in the ER, including immunoglobulin-binding protein (BiP), a heat shock protein (Hsp 70) family chaperone, Calnexin (CNX), and Calreticulin (CRT), play important roles in ER QC. CNX and CRT mediate folding of Asn-linked glycoproteins trafficking through the ER (Leach and Williams, 2003). Heat shock 70-kDa proteins transiently bind to their client proteins through an ATP hydrolysis and ATP rebinding cycle (Mayer and Gierasch, 2019). This cycle is regulated by DnaJ/Hsp40 (heat shock protein 40) proteins, which contain J-domain a ∼70 amino acid signature sequence region through which they bind to Hsp70s (Kampinga and Craig, 2010).

## Endoplasmic Reticulum Stress and Male Reproductive Development

Plants, being sessile, cannot move to avoid adverse effects of heat waves that are increasing in duration and frequency due to current global climate change conditions. Though heat stress adversely impacts all plant growth stages, the plant reproductive development remains the most vulnerable stage of the life cycle. This vulnerability at the reproductive stage leads to a significant reduction in seed set and crop yields. The pollen development and pollination are particularly vulnerable to heat stress events as elevated temperatures during pollen development can trigger pollen abortion (Reiu et al., 2017; Begcy et al., 2019; Lohani et al., 2020, 2021). Recent investigations have uncovered the crucial role of the UPR for ensuring normal pollen development and successful fertilization (Deng et al., 2013, 2016; Fragkostefanakis et al., 2016). An active UPR pathway is required to meet the high demands of secretory proteins during normal development even in the absence of exogenous stresses (Deng et al., 2013). The knockout mutations in the UPR signaling pathway genes result in pollen developmental abnormalities primarily resulting in male sterility (Table 1). In this review, we focus on the ER stress pathways concerning pollen vulnerability to heat stress conditions.

**TABLE 1:** A summary of Arabidopsis ER stress response genes and roles as determined by fertility phenotypes in gene knockout mutants.

Gene name	Arabidopsis Gene id	Gene Product Localization	Arabidopsis Mutant	Pollen Development Phenotype at Normal Temperature	Pollen Development Phenotype under Heat Stress	References
bZIP28; BASIC REGION/LEUCINE ZIPPER MOTIF 28	AT3G10800	ER membrane, Cytoplasm, Nucleus	*bzip28 bzip60* double mutant	Normal fertility	Reduced fertility, silique lengths in *bzip28 bzip60* double mutant plants were largely reduced compared with the wild-type plants	Zhang et al., 2017
bZIP60; BASIC REGION/LEUCINE ZIPPER MOTIF 60	AT1G42990	ER membrane and nucleus				

IRE1a; INOSITOL REQUIRING 1A	AT2G17520	ER membrane	*ire1a ire1b* double mutant	Normal viable pollen	Temperature-sensitive male sterility, improper deposition of pollen coat materials possibly due to tapetal defects, shortened siliques generally devoid of seeds	Deng et al., 2016
IRE1b; INOSITOL REQUIRING 1B	AT5G24360					

CNX1; CALNEXIN HOMOLOG 1	AT5G61790	ER membrane	*cnx1 crt1 crt2 crt3*	Diverse effect on pollen viability and pollen tube growth, leading to a significant reduction pollen mediated transmission		Vu et al., 2017
CRT1; CALRETICULIN1	AT1G56340	ER and vacuole membrane, secretory vesicles				
CRT2; CALRETICULIN2	AT1G09210	ER and vacuole membrane, secretory vesicles	*cnx1 cnx2 crt1 crt2 crt3*	Lethal—no pollen mediated transmission		

CRT3; CALRETICULIN3	AT1G08450	ER lumen				
BiP1; ER localized member of HSP70 family	AT5G28540	ER lumen and nucleus	*bip1 bip2* double mutant	Significant reduction in pollen tube growth activity		Maruyama et al., 2010

BiP2; LUMINAL BINDING PROTEIN	AT5G42020	ER lumen and nucleus	*bip1 bip2 bip3* triple mutant	Lethality of pollen due to defects in mitosis1, bicellular stage that contained one or two abnormal microspores with one nucleus		Maruyama et al., 2014
BiP3; HSP70 FAMILY PROTEIN	AT1G09080	ER lumen and nucleus				

SHD/HSP90; SHEPHERD, HEAT SHOCK PROTEIN 90-7	AT4G24190	ER lumen	*shd*	Defects in pollen−tube elongation or penetration into the style	Increased the severity of the defects	Ishiguro et al., 2002

ERdj2A/SEC. 63-1;J-Domain protein	AT1G79940	ER membrane	*aterdj2a-1, aterdj2a-2*	Defects in pollen germination but not pollen development		Yamamoto et al., 2008

ERdj3A/TMS1; THERMOSENSITIVE MALE STERILE 1	AT3G08970	ER lumen	*tms1-1*	The fertility of tms1-1 plants was slightly affected, with some ovules in the lower part of the siliques unfertilized	Greatly retarded pollen tube growth in the transmitting tract, resulting in a significant reduction in male fertility	Yang et al., 2009

ERdj3B; J-Domain protein	AT3G62600	ER lumen	*erdj3b*	Normal flower development and fertility	Produced few seeds at high temperatures due to anther development defects, abnormal enlargement of tapetum cells with vacuolated and aborted microspores, defective pollen release from the anthers	Yamamoto et al., 2020
			*atp58ipk aterdj3b*	Defects in male gametophyte		Yamamoto et al., 2008

Sec62; protein with similarity to yeast Sec62p.	AT3G20920	ER membrane	*atsec62* (T-DNA and amiRNAi)	Smaller and round depressed pollens, defects in pollen development, smaller, aborted, and lesser number of siliques		Hu et al., 2020;
			*atsec62*	Aborted and mostly empty siliques, delayed anther and pollen development, less pollen released from mutant anthers and reduced pollen germination	Pollen hardly germinated	Mitterreiter et al., 2020

PDI9; PROTEIN DISULFIDE ISOMERASE 9	AT2G32920	ER lumen	*pdi9*	Normal viable pollen	Disruptions in the reticulated pattern of the exine and an increased adhesion of pollen grains	Feldeverd et al., 2020
PDI10; PROTEIN DISULFIDE ISOMERASE 10	AT1G04980	ER lumen	*pdi9 pdi10* double mutant	Normal viable pollen	Completely lost exine reticulation	

POD1; POLLEN DEFECTIVE in GUIDANCE 1	AT1G67960	ER lumen	*pod1*	Pollen tubes fail to target the female gametophyte, defective in micropylar pollen tube guidance leading to zygotic lethality		Li et al., 2011

UTR1, UDP-GALACTOSE TRANSPORTER 1	AT2G02810	ER and golgi membranes	*utr1 utr3* double mutant	Abnormalities in both male and female germ line development, haploid atutr1 atutr3 combination is a fully penetrant lethal mutation for the male gametophyte and is partially penetrant for the female gametophyte		Reyes et al., 2010

UTR3, UDP-GALACTOSE TRANSPORTER 3	AT1G14360					
STT3a; STAUROSPORIN AND TEMPERATURE SENSITIVE 3-LIKE A	AT5G19690	ER membrane	*stt3a-1 stt3b-1* double mutant	Gametophytic lethal		Koiwa et al., 2003

SERK1; SOMATIC EMBRYROGENESIS RECEPTOR-LIKE KINASE 1	AT1G71830	ER and cell membrane	*serk1 serk2*	Completely male sterile due to a failure in tapetum specification, double mutant anthers lack development of the tapetal cell layer leading to the microspore abortion and male sterility		Albrecht et al., 2005; Colcombet et al., 2005

SAR1; SECRETION ASSOCIATED RAS 1	AT1G56330	ER-, COPII vesicle coat and golgi apparatus	*sar1b sar1bsar1c* double mutant	Malfunctioning tapetum, leading to male sterility. Microspores in sar1b pollen sacs started to degenerate. The plasma membrane (PM) of microspores in sar1b pollen sacs was detached from the cell wall, and at anther dehiscence, sar1b pollen sacs contained only a pile of cellular debris Microspores aborted at anther developmental stage 10, arrest of pollen development at Pollen Mitosis I		Liang et al., 2020

PDR2, PHOSPHATE DEFICIENCY RESPONSE 2	AT5G23630	ER membrane	*mia* mutants	Male gametogenesis impaired anthers (*mia*) show severe reduction in fertility. Mutant microspores fail to separate from tetrads and fragile pollen grains with an abnormal morphology and altered cell wall structure.		Jakobsen et al., 2005

AEP1; ASPARAGINYL ENDOPEPTIDASE 1	AT2G25940	Protein storage vacuole, Vacuole	β*vpe*	Abnormal degradation of the tapetum, incomplete pollen cytoplasm development, with few oil bodies and an indistinct generative cell. Some of the pollen grains were shrunken and abnormally shaped, immature pollen still contained numerous small vacuoles		Cheng et al., 2020

CEP1; CYSTEINE ENDOPEPTIDASE 1	AT5G50260	ER and vacuole	*cep1*	Aborted tapetal PCD, reduced male fertility due to impaired pollen development and abnormal pollen exine		Zhang et al., 2014

RBOHE; Riboflavin Synthase-Like Family Protein	AT1G19230	Multi pass membrane protein	*rbohe-2*	Delayed degeneration of tapetum, reduced pollen viability, abnormal pollen grain shape and exine layer		Xie et al., 2014

RBOHJ; RESPIRATORY BURST OXIDASE HOMOLOG J	AT3G45810	Multi pass membrane protein	*rbohH rbohJ* double mutant	Pollen tip growth severely impaired due to impaired ROS accumulation		Kaya et al., 2014

RBOHH; RESPIRATORY BURST OXIDASE HOMOLOG J	AT5G60010	Multi pass membrane protein				

## Endoplasmic Reticulum Stress Pathways and Pollen Development

**FIGURE 2:**
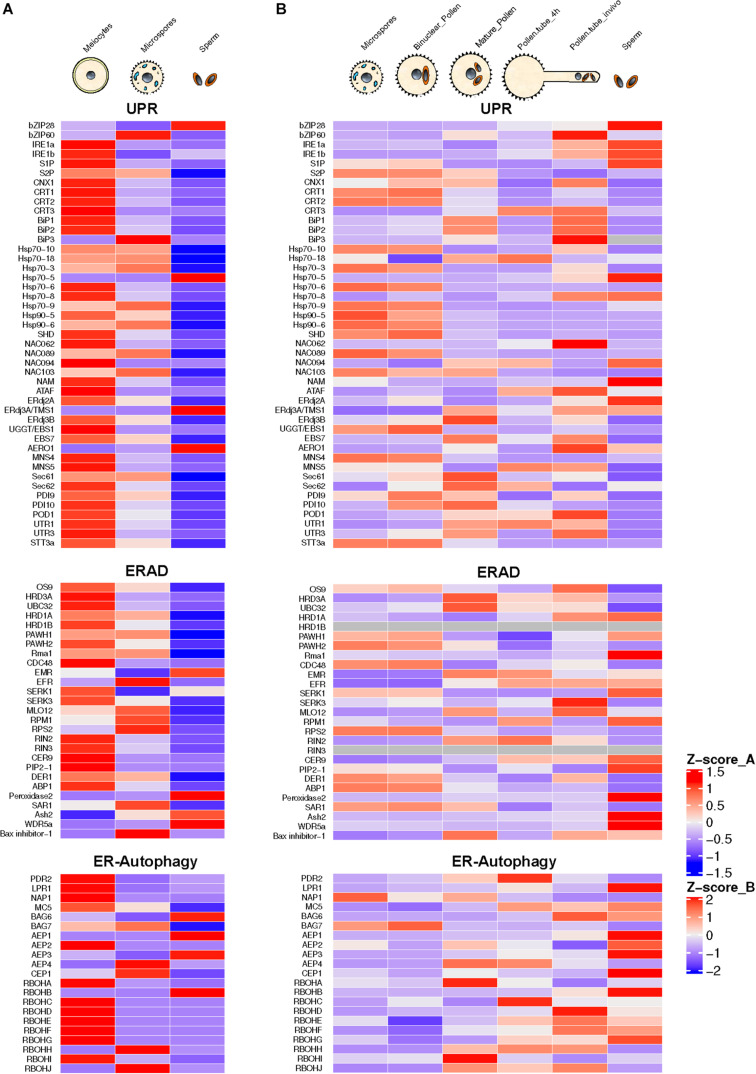
Annotation and expression of ER stress component genes in Arabidopsis developing pollen, pollen tubes, and sperm cells. The data were compiled using the gene expression obtained from RNA-seq based analysis **(A)** or microarrays **(B)**. RNA-seq data sets from previously published literature were downloaded from the NCBI Sequence Read Archive database. For Microarray data, the expression values were downloaded from the Arabidopsis Heat Tree Viewer (http://arabidopsis-heat-tree.org/). The gray color in the heatmap denotes missing values. Full details available in Supplementary File 1.

The process of pollen development from meiocytes to microspores involves intense protein biosynthesis and trafficking of secretory proteins through ER and Golgi apparatus. Arabidopsis mutants for genes involved in the ER to Golgi trafficking exhibit male sterility phenotype (Conger et al., 2011; Tanaka et al., 2013; Deng et al., 2016). High requirement for secretory proteins in developing pollen triggers ER stress constitutively. This constitutive functioning of UPR has been reported to be essential for pollen development (Deng et al., 2016). This conclusion is also supported by the presence of spliced forms of bZIP60 in *Arabidopsis* male reproductive tissues under normal conditions (Iwata et al., 2008; Deng et al., 2016). The transcriptome-wide mining of male meiocytes and microspores from *Arabidopsis* plants growing under normal conditions reveals highly elevated expression of most of the ER stress and UPR component genes (Figure 2). Many of these genes show the highest expression levels in diploid meiocytes (microspore mother cells) undergoing meiosis. ER stress component genes highly expressed in the meiocytes belong to UPR, ERAD, and the ER autophagy programs. Among most conspicuous ER phagy genes are those encoding members of Respiratory Burst Oxidases Homolog (RBOH) family, which comprises 10 NADPH oxidase genes in Arabidopsis (Chang et al., 2016). Seven out of 10 gene members show highly elevated expression in meiocytes. The majority of the knockout mutants of *Arabidopsis* ER stress-related genes involved in UPR, ERAD, and ER-autophagy processes show loss of fertility phenotypes in plants grown under non-stressed conditions (Table 1). These observations highlight the essential role of ER homeostasis in permitting normal pollen development.

ROS generated by these NADPH-dependent oxidases (Nox) complex control various developmental processes including programmed tapetal cell death and is an essential component of developing pollen–tapetum interaction network (Xie et al., 2014). Other ER autophagy-related genes showing a high level of expression in diploid meiocytes include Metacaspase 5 (MC5) and Bcl-2-associated athanogene 7 (BAG7). MC5 and BAG7 are involved in the programmed cell death (PCD) network. Metacaspases are cysteine proteases involved in PCD that are distantly related to animal caspases (Kørner et al., 2015). MC5 has been shown as a positive regulator of ER stress-induced PCD (Sobri and Zulfazli, 2016). AtBAG7 is a member of the Arabidopsis BAG family encoding plant homologs of mammalian regulators of apoptosis (Li et al., 2017b). These ER-localized proteins play a crucial role in maintaining UPR in response to external stresses (Li et al., 2017b; Nawkar et al., 2018).

## Endoplasmic Reticulum Stress During Post-Meiotic Development and Pollen Tube Growth

In post-meiotic stages of pollen developmental progression, several ER stress component genes show expression at the unicellular microspore and bicellular stage. These include genes encoding BiPs, both IRE1 isoforms (IRE1a and IRE1b), CRT, CNX, site-specific proteases (S1P and S2P), and NAC family of membrane-bound transcription factors.

Another noticeable feature of the developing pollen transcriptome is high-level expression of *ERAD* genes. Gene encoding UBC32, a ubiquitin-conjugating enzyme (E2) localized in the ER membrane, is expressed constitutively in the male meiocytes. Its expression, although much lower in microspores, shows an increase during the pollen development. The Arabidopsis genome contains five J-domain encoding genes (AtERdj3A, AtERdj3B, AtP58^*IPK*^, AtERdj2A, and AtERdj2B) that encode Hsp40 family co-chaperones of Hsp70. The Arabidopsis Thermosensitive Male Sterile 1 (TMS1) encoding a J-domain protein identical to AtERdj3A plays a significant role in determining thermotolerance of pollen and vegetative tissues (Ma et al., 2015). Arabidopsis plants carrying a knockout mutation in TMS1 grown at 30°C were reported to show a significant decrease in male fertility resulting from retarded pollen tube growth in the stylar transmitting tract (Yang et al., 2009). Recently, Yamamoto et al. (2020) reported that a second ER-resident Arabidopsis J-domain protein, AtERdj3B, also plays a critical role in another development at elevated temperatures. The *erdj3b* mutant showed a significantly reduced seed set at an elevated temperature of 29°C. This reduced seed set phenotype could be rescued in mutants by introducing *ERDJ3B* gene expressing under its promoter. Interestingly, this defect could be rescued by overexpression of *ERDJ3A* gene regulated by the *ERDJ3B* promoter. The *erdj3b* mutant plants grown at 29°C revealed collapsed pollen with abnormalities in their pollen coats. Furthermore, the authors addressed whether pollen-coat abnormality in erdj3 at elevated temperatures is caused by the effect of the mutation in tapetal cells. The transformation of *erdj3b* mutant plants with *ERDJ3B* gene expressing under a tapetum-specific promoter led to partial suppression of the reduced seed set phenotype in mutant plants growing at 29°C. Interestingly, this study could not observe fertility defects in *erdj3a-1* or *erdj3a-2* mutants grown at 29°C. It was further proposed that among ER-localized three J proteins, heat stress-sensitive fertility defect results only from defective interaction of only EEdj3B with BiP. Three Hsp70 chaperone proteins (BiP1, BiP2, and BiP3) are localized in ER of *Arabidopsis thaliana* (Yamamoto et al., 2008; Ma et al., 2015). BiP1 and BiP2 are 99% identical and have been reported to be expressed ubiquitously. Interestingly, BiP1 and BiP2 expression is significantly upregulated in Arabidopsis meiocytes (Figure 2). BiP3 that shows less identity with the other two paralogs is expressed only under ER stress conditions (Maruyama et al., 2015). Maruyama et al. (2010) have shown that the *Arabidopsis bip1/bip2* double mutant shows normal pollen viability but retarded pollen tube growth both *in vitro* and *in vivo*. Since the secretion of cell wall proteins is crucial for pollen tube growth, the reduced BiP level led to retarded pollen tubes growth rates due to decline in protein translocation, protein folding, and ER QC activities.

Recently, Poidevin et al. (2020) used a Riboprofiling technique to unravel the effect of heat stress on transcriptome and translatome of mature and *in vitro* germinated Arabidopsis pollen grains. Riboprofiling (Ribo-seq) allows accurate comparison of cellular transcriptome with translatome (Ingolia et al., 2009; Hsu et al., 2016). Riboprofiling data showed transcriptional and translational level upregulation of DNA-J chaperones and ER stress related in germinated pollen, induced by the heat stress. These upregulated genes include Hsp70 BiPs (BiP1, BiP2, and BiP3), the DNAJ chaperones (ERDJ3A, ERDJ3B, and P581PK), Calnexin and Calreticulin (CNX1, CRT1a, and CRT1b), and proteins involved in ERAD pathway such as DER1 and DER2.1. The key transcription factors, bZIP28, bZIP60, and NF-YC2, are transcribed and translated in pollen tubes (Poidevin et al., 2020). The authors conclude from their data that the Arabidopsis pollen can respond to heat stress by enhancing the expression of thermotolerance genes. However, their data are based on transcriptome and translatome profiling of *in vitro* germinated pollen grains. The transcriptional repertoire of pollen tubes penetrating the stigma and styler tissues is very different from that of *in vitro* germinated pollen tubes with *in vivo* growing tubes expressing a substantially larger fraction of the genome (Qin et al., 2009). The analysis of transcriptome data of Arabidopsis pollen tubes growing *in vivo* shows default upregulation of expression of ER stress genes in the absence of external stress (Figure 2).

The transcriptional activity of ER stress-related genes in the pollen germinating *in vitro* is comparable to mature ungerminated pollen with no significant change in expression levels. However, the pollen tubes growing in the styler transmitting tissues show significant upregulation of several ER stress component genes such as bZIP60, both isoforms of IRE1, S2P, NAC062, J-domain protein ERdj2a, and AERO1 (ER oxidoreductin 1). The observed stark differences in the gene expression patterns in pollen tubes growing *in vitro* or *in vivo* are predictable as pollen tubes attain about 135 μm length *in vitro* (Dickinson et al., 2018), while pollen tubes growing *in vivo* have to traverse stigma/style length. Rapidly growing pollen tubes show high trafficking with secretory vesicles providing membrane components (Campanoni and Blatt, 2007). High demands for secretory proteins likely trigger UPR in the pollen tubes. Interestingly, Arabidopsis mutants for many UPR genes display pollen germination and pollen tube growth defects (Ishiguro et al., 2002; Yamamoto et al., 2008; Maruyama et al., 2010; Deng et al., 2013).

The ERAD component genes expressing highly in pollen tubes include genes encoding OS9, HRD3A, PAWH1, Rma1, MLO12, CER9, EBS7, and Bax-inhibitor-1. High ER-associated protein degradation appears to be a hallmark of rapidly elongating pollen tubes. ERAD involves modifying target unfolded/misfolded proteins with ubiquitin, removal from the ER, followed by degradation by the cytoplasmic 26S proteasome (Preston and Brodsky, 2017). AtOS9 is an Arabidopsis homolog of mammalian ER luminal lectin OS9 with binding specificity for asparagine-linked glycan on misfolded proteins. An interesting feature of the pollen tube transcriptome is a high expression of ER phagy-related genes encoding BAG6, MC5, AEP1, RBOHH, and RBOHJ (Respiratory Burst Homologs). RBOHH and RBOHJ encode NADPH oxidases containing Ca^2+^ binding EF-hand motifs and possessing Ca^2+^-induced ROS production activity. While Arabidopsis single mutants, *rbohH* and *rbohJ*, attain pollen tube growth comparable to wild type, the double mutant showed severe impairment of pollen tube growth (Kaya et al., 2014). Also, *in vitro* grown pollen tubes of the *rbohH* and *rbohJ* double mutants rupture easily (Boisson-Dernier et al., 2013).

## Expression of ER Stress-Related Genes in Sperm Cells

Pollen is largely made up of vegetative cell that forms a pollen tube, which acts as a conduit to transmit male germline, the non-motile sperm cells, into the female gametophyte to execute double fertilization, a defining feature of flowering plants (Singh and Bhalla, 2007). The male germline is initiated by asymmetric division of the microspore leading to the formation of much smaller generative cell enveloped within the larger vegetative cell. The generative cell divides once again to produce two sperm cells required for double fertilization (Russell and Jones, 2015). In the mature pollen, sperm cells may comprise much less than 1% of the pollen volume (Russell and Strout, 2005; Russell and Jones, 2015). For a long time, the inconspicuous generative and sperm cells were considered passive carriers of male genetic lineage. However, it was later shown that both these cells are largely transcriptionally and translationally distinct from much larger vegetative cells (Xu et al., 1999; Singh and Bhalla, 2007). Transcriptomic analysis using either microarrays or RNA-seq approaches have highlighted the highly divergent nature of gene expression in generative and sperm cells compared to that of the vegetative cells (Okada et al., 2007; Singh et al., 2008; Russell et al., 2012; Russell and Jones, 2015). A survey of ER stress-related gene expression in Arabidopsis sperm isolated from mature pollen reveals constitutive expression of several ER stress-related genes relating to UPR, ERAD, and ER phagy with remarkably high expression of UPR genes encoding bZIP 28, ERdj3A/TMS1, and AERO-1. ERAD-related genes showing significant expression in sperm cells include SERK1, Peroxidase 1, Ash2, and WDR5a. ER autophagy-related genes with significant expression in sperm cells include BAG6, AEP1, AEP3, and RBOHB. While the data from microarray and RNA-seq experiments cannot be compared directly, there appears to be a good overall cross-platform concordance particularly among genes showing high expression levels. The quantitative expression pattern of ER stress related in sperm cells is quite distinct from that of total pollen (Figure 2). However, it remains an open question whether the pattern of ER stress gene expression show further changes in sperm cells following heat stress or due to pollen tube growth in female tissues.

## Future Perspectives

Despite an increasing number of publications on plant reproduction and ER stress response in recent years reporting intriguing findings, there remain open questions about the activation of ER stress and the role this response plays in protecting pollen development and pollination processes from detrimental effects of excessive heat exposure. The cellular trigger for high constitutive activity of ER stress-related genes in diploid meiocytes warrants further investigation. An intriguing possibility is the potential role of cellular hypoxia in triggering UPR in meiocytes. Studies on animal systems have revealed that the activation of UPR is an adaptive response to hypoxic stress (Bartoszewska and Collawn, 2020). Earlier, Kelliher and Walbot (2012) have proposed that meiotic fate in the archesporial cells in the immature anthers is triggered by hypoxia. Future investigations can focus on the potential crosstalk between hypoxia conditions in the anther cavity and triggering of UPR in resident meiocytes.

It has been recognized that pollen development is one of the situations where high demands for secretion triggers ER stress under normal conditions without externally imposed stress (Howell, 2017). Thus, it can be postulated that the protein homeostasis maintained by enhanced protein folding capacity allows normal pollen development to proceed. There is no evidence for the direct interaction between the pollen ER stress response and the transcription factors and downstream pathways linked to regulation of cell fate determination and developmental progression. The constitutive ER stress response leading to near-capacity functioning of the ER protein folding and trafficking machinery likely diminishes ER’s adaptive capacity to adjust to external stresses, resulting in the high sensitivity of pollen to heat stress events. Thus, future studies can be expected to focus on investigating the overexpression of key ER signaling components and chaperones as a tool to enhance pollen thermotolerance. This would open new opportunities for engineering crop plants that can offer yield stability in the face of increased frequency of heat waves with crops getting exposed to extreme temperature events.

## Author Contributions

NL analyzed the sequencing data. MS conceived the research. MS and PB drafted the manuscript. All authors contributed to the article and approved the submitted version.

## Conflict of Interest

The authors declare that the research was conducted in the absence of any commercial or financial relationships that could be construed as a potential conflict of interest.
