# Supplementary material for: The Role of Endoplasmic Reticulum Stress Response in Pollen Development and Heat Stress Tolerance
Source: Front Plant Sci. 2021 Apr 14;12:661062. doi: 10.3389/fpls.2021.661062 (PMC8079734; doi:10.3389/fpls.2021.661062)
Supplement: Supplementary file 1 [file Data_Sheet_1.docx]

**RNA Seq DATA**

**Table 1: Details of the RNA-Seq libraries used in Figure 2.**

| **Tissue** | **Replicate** | **Sequencing** | **Lib-type** | **SRA** | **BioProject** |
| --- | --- | --- | --- | --- | --- |
| Male meiocytes | rep_1 | Illumina HiSeq 2000 | Paired | SRR8352562_1.fastq.gz | PRJNA510650 |
|  |  |  |  | SRR8352562_2.fastq.gz |  |
|  | rep_2 |  | Paired | SRR8352561_1.fastq.gz |  |
|  |  |  |  | SRR8352561_2.fastq.gz |  |
|  |  |  |  |  |  |
| Microspores | rep_1 | Illumina HiSeq 2500 | Single | SRR10066374.fastq.gz | PRJNA563854 |
|  | rep_2 |  | Single | SRR10066375.fastq.gz |  |
| Sperm Cells | rep_1 | Illumina HiSeq 2500 | Paired | SRR7945266_1.fastq.gz | PRJNA494054 |
|  |  |  |  | SRR7945266_2.fastq.gz |  |
|  | rep_2 |  | Paired | SRR7945267_1.fastq.gz |  |
|  |  |  |  | SRR7945267_2.fastq.gz |  |
|  | rep_3 |  | Paired | SRR7945268_1.fastq.gz |  |
|  |  |  |  | SRR7945268_2.fastq.gz |  |

**Method:** RNASeq data sets from previously published literature were downloaded from NCBI Sequence Read Archive database (Table 1). Transcript expression was quantified using Kallisto v0.44.0 and read abundance was expressed as Transcripts Per Kilobase Million (TPM). Heat maps were drawn by using ComplexHeatmap package to visualise the expression of the selected genes. The scale bar represents the Z-score (scaled TPM values).

**MICROARRAY DATA**

For Microarray data the expression values were downloaded from Arabidopsis Heat Tree Viewer (<http://arabidopsis-heat-tree.org/>). The reproductive expression series is compiled from several independent microarray experiments. To help make them comparable, they have been processed together. All have between 2 and 4 replicates for each sample. The details about each are located within their respective papers. Citations:-Boavida, Leonor C et al. “Whole Genome Analysis of Gene Expression Reveals Coordinated Activation of Signaling and Metabolic Pathways During Pollen-Pistil Interactions in Arabidopsis..” Plant physiology 155.4 (2011): 2066–2080. Borges, Filipe et al. “Comparative Transcriptomics of Arabidopsis Sperm Cells..” Plant physiology 148.2 (2008): 1168–1181. Web.-Honys, David, and David Twell. “Transcriptome Analysis of Haploid Male Gametophyte Development in Arabidopsis..” Genome biology 5.11 (2004): R85. Qin, Yuan et al. “Penetration of the Stigma and Style Elicits a Novel Transcriptome in Pollen Tubes, Pointing to Genes Critical for Growth in a Pistil..” PLoS genetics 5.8 (2009): e1000621.
